# Treatment of the Apparently Drowned

**Published:** 1859-01

**Authors:** 


TREATMENT OF THE APPARENTLY DROWNED.—The following instructions for the resuscitation of the apparently drowned have been extensively circulated by the National Lifeboat Institution throughout the kingdom and our colonies. They contain the latest discoveries on this important subject, the institution having taken great pains to obtain the opinion of the medical profession generally both at home and on the continent on the comparative merits of this and the antiquated plan hitherto adopted in this country. Of the 300 medical men, some of them representing medical bodies, who gave the society their opinion on the subject, 254 were entirely in favor of the new method, 10 were on the side of the old plan, and 36 were undecided. It should be stated that the leading principles of the instructions are those of the eminent physiologist, the late Dr. Marshall Hall:—"1. Treat the patient instantly on the spot, in the open air, exposing the face and chest to the breeze, (except in severe weather.) 2. To clear the throat, place the patient gently, face downward, with one wrist under the forehead, in which position all fluids will escape by the mouth, and the tongue itself will fall forward, leaving the entrance into the windpipe free. Assist this operation by wiping and cleansing the mouth. If there be breathing—wait and watch; if there be no breathing, or if it fail, then—to excite respiration—3. Turn the patient well and instantly on the side; and 4. Excite the nostrils with snuff, hartshorn, volatile salts, or the throat with a feather, etc., and dash cold water on the face, previously rubbed warm. If there be no success, lose not a moment, but instantly, to imitate respiration—5. Replace the patient on the face, raising and supporting the chest well on a folded coat or other article of dress. 6. Turn the body very gently on the side and a little beyond, and then briskly on the face, alternately; repeating these measures deliberately, efficiently, and perseveringly about fifteen times in the minute, or every four seconds, occasionally varying the side. (By placing the patient on the chest it is compressed by the weight of the body, and expiration takes place; when turned on the side this pressure is removed, and inspiration occurs.) 7. On each occasion that the body is replaced on the face make uniform but efficient pressure with brisk movement, on the back between and below the shoulder-blades or bones on each side, removing the pressure immediately before turning the body on the side. (The first measure increases the expiration, the second commences inspiration.) The result is respiration or natural breathing; and, if not too late, life. 8 After respiration has been restored, promote the warmth of the body by the application of hot flannels, bottles, or bladders of hot water, heated bricks, etc., to the pit of the stomach, the armpits, between the thighs, and to the soles of the feet. To induce circulation and warmth—9. During the whole time do not cease to rub the limbs upward, with firm grasping pressure and with energy, using handkerchiefs, flannels, etc. (By this measure the blood is propelled along the veins toward the heart.) 10. Let the limbs be thus warmed and dried, and then clothed, the by-standers supplying the requisite garments.

"Cautions.—1. Send quickly for medical assistance and for dry clothing. 2. Avoid all rough usage and turning the body on the back. 3. Under no circumstances hold up the body by the feet; 4. Nor roll the body on casks; 5. Nor rub the body with salt or spirits; 6. Nor inject tobacco smoke or infusion of tobacco. 7. Avoid the continuous warm bath. 8. Be particularly careful to prevent persons crowding around the body.

"General Observations.—On the restoration to life a teaspoonful of warm water should be given, and then, if the power of swallowing has returned, small quantities of wine, or brandy and water warm, or coffee. The patient should be kept in bed, and a disposition to sleep encouraged. The treatment recommended should be persevered in for a considerable time, as it is an erroneous opinion that persons are irrecoverable because life does not soon make its appearance, cases having been successfully treated after persevering several hours."—*London Times.*

